# Piezoelectric and dielectric properties of Bi_3_TiNbO_9_ prepared by hot pressing from powders activated using the serial dilution method

**DOI:** 10.1038/s41598-020-78826-w

**Published:** 2020-12-17

**Authors:** A. I. Spitsin, A. A. Bush, K. E. Kamentsev

**Affiliations:** MIREA – Russian Technological University (RTU MIREA), Moscow, Russia 119454

**Keywords:** Ferroelectrics and multiferroics, Electronic devices

## Abstract

Bi-based layer structure ferroelectrics are the most promising compounds for the fabrication of high-temperature piezoelectric materials. Studies aiming to develop and optimize the techniques to produce efficient high-density piezoelectric ceramics, and to investigate the effects of ceramics production conditions on their structure and functional properties, have become high-priority objectives of modern piezo-engineering. We applied ultra high dilution (UHD) technology to pre-treat Bi_3_TiNbO_9_ powders and used hot pressing to prepare perovskite-layer structured ceramic specimens. Main characteristics of the synthesized piezoelectric ceramic specimens (the dimensions of the Bi_3_TiNbO_9_ orthorhombic unit cell, dielectric permittivity, dielectric loss, piezoelectric coefficient d_33_ and pyroelectric coefficient p^σ^) and their temperature-dependent variations were studied using piezoelectric, dielectric, and pyroelectric measurements. X-ray diffraction studies demonstrated that the prepared ceramics were single phased, and highly textured, as their plate-like crystallites were preferentially aligned perpendicularly to the pressure axis on hot pressing. For d_33_, an increase in values of more than 20% was found for samples obtained using a combined modification of the UHD technology and hot pressing (12 pC/N) relative to intact samples, and more than two times relative to unmodified Bi_3_TiNbO_9_ ceramics (6 pC/N). Due to their characteristics, the obtained ceramics are promising materials for high-temperature applications; of particular interest is potential use, as electroacoustic transducers and sensors for operation at high temperatures. Thus, the UHD technology can modify the properties of ceramics and is relatively easy to implement. This makes it attractive for use in various fields of science and technology.

## Introduction

The wide use of piezoelectric devices in aircraft, aerospace and atomic energy industries has raised interest and demand for piezoelectric materials with parameters making them suitable for operation at elevated temperatures^1,2^. To date a vast variety of electromechanical transducers, such as sensors, actuators, ultrasonic irradiators, receivers, etc., that can be used at extreme conditions have been designed^1–4^. The expansion of the scope and areas of application for piezoelectric materials has resulted in higher operational requirements for such materials, as well as in the need for developing new piezoelectric ceramics with broadly varying dielectric and electromechanical characteristics optimized for different applications. Manufacture of piezoelectric ceramics operational at high temperatures (T > 700 °C)^1,2^ is an essential area of piezoelectric technology for the applications carried out in severe environments, such as space exploration or operational testing of engines, turbines, etc. However, the majority of well-known and commonly used piezoelectric materials cannot furnish stable operation at high temperatures (T > 300 °C) due to their insufficiently high Curie temperature T_c_, and therefore the ferroelectric-to-paraelectric transition occurring at T > T_c_.

The fabrication of high-temperature piezoelectric materials is based on the use of ferroelectrics with high T_c_. The most promising of these are ferroelectric Bi-containing compounds [Bi_2_O_2_][A_n−1_B_n_O_3n+1_] having perovskite layer structure (Bi-based layer structure ferroelectrics—BLSF, or so-called Aurivillius phases—AP), the Curie temperature of which can exceed 900 °C (1173 К)^5–11^. Bismuth titanate niobate Bi_3_TiNbO_9_ (BTN) is a member of the family of BLSF, with A = Bi, B = Ti, Nb and the number of BO_6_ octahedra in the perovskite layer along the packing between adjacent Bi_2_O_2_ layers being n = 2, and with BTN having the highest Curie temperature T_c_ among the BLSF family (1223 К)^7^. It has been found that spontaneous polarization **P**_s_ is perpendicular to the pseudo-tetragonal c axis (along the ***a ***axis) when n is an even number (space group B2cb), as required for phase symmetry^9–11^.

BLSF materials, due to their high Curie temperatures and resistance to fatigue during the transition in spontaneous polarization, have recently attracted vivid interest in terms of possible applications in piezoelectric engineering and information technology, as promising sources for high-temperature piezotransducers capable of operating under extreme conditions, as well as non-volatile random access memory (NvFERAM)^11–14^.

The possibilities to synthesize and exploit such high-temperature piezoelectric ceramics have been limited by their characteristic elevated porosity (10–25%) associated with poor compactibility during the sintering of petal-like crystallites common in BLSF^8,15–21^. Also, problems with the synthesis of high-temperature BLSF piezoelectric ceramics include their comparatively high conductivity and depolarization at T > 870 К^8,15–23^. BTN specimens manufactured using the standard ceramics technology typically have low d_33_ values (≤ 7 pC/N)^15,16,18^ attributed to their high porosity, and disordered microstructure. The hot pressing method, where the largest grain planes align themselves perpendicularly to the pressure applied, enables producing high-density perovskite-layer anisotropic ceramics^8,16,17,24–27^.

For these reasons, studies aiming to develop and optimize the techniques to produce efficient high-density BLSF piezoelectric ceramics, and to investigate the effects of ceramics production conditions on their structure and functional properties, have become high-priority objectives of modern piezo-engineering. The serial ultra high dilution (UHD) process seems to have potential to cause an effect on the parameters of high-density ceramics, improving the production techniques, and expanding the applications of devices based on such materials. It has been previously shown that process of multiple sequential dilution of starting materials leads to a significant change in the properties of the resulting solutions (structuredness, kinetic and thermodynamic characteristics) in comparison with solutions that have not been subjected to such treatment^28,29^. When added to the original substance UHD solutions may affect its properties as well^30^.

A feature of this technique is a nonlinear decrease in concentration of the starting material during multiple sequential dilutions, which ultimately leads to the formation of nanostructures^31^. It is known that nanostructures have unique physical^32^ and biological^33^ properties that are different from the solvent and the starting material. Thus, we hypothesized that such nanostructures could affect properties of the materials used for ceramics manufacturing.

In this study, we used a typical perovskite-layer structured Bi_3_TiNbO_9_-based high-temperature piezoelectric ceramics (with the usual parameters in its class) for a general assessment of the applicability of the UHD technology to materials used in industry. This is the first presentation of results from a study evaluating the effects of the UHD technology on the parameters of high-temperature ceramics.

## Materials and methods

### Preparation of mixture components

High purity starting raw materials were obtained from Elpa Research Institute, Moscow, Russia. Bi_2_O_3_ (≥ 99% purity), Nb_2_O_5_ (≥ 98% purity), and TiO_2_ (≥ 99% purity) were mixed at a 3:1:2 molar ratio corresponding to the chemical composition of Bi_3_TiNbO_9_.

The mixture was homogenized in distilled water using an HD/01 attritor mill from Union process (USA) which provides high degree of powder fineness and almost does not contaminate the product with iron or other substances, as the milling process uses zirconium oxide balls of high hardness and low friability. As a result, the size of the stock component particles was reduced to a few micrometers.

### The synthesis of BTN powders

This was performed using the solid-state reaction method by annealing the homogenized Bi_3_TiNbO_9_ mixtures at 1273 К for 6 h. Two annealing runs were carried out, with the synthesis product grinding in between the runs.

As a result, yellowish-brown polycrystalline Bi_3_TiNbO_9_ specimens, uniform in appearance, were obtained. The synthesized material was milled in the attritor and then dried. The obtained Bi_3_TiNbO_9_ powders with a specific surface area S_sp_ of 3500 to 6000 cm^2 ^g^−1^ were portioned into 4 batches.

### Modification of BTN powders

UHD technology is usually applied via multiple sequential dilutions of substances in water–ethanol solutions. However, layered perovskite-like oxides are insoluble in aqueous ethanol. Therefore, to increase their solubility, initial concentration reduction was performed with the use of technology of trituration of powders with lactose which is soluble in aqueous ethanol (described below, see stage 1).

The modification by special processing of Bi_3_TiNbO_9_ powders was performed in 3 stages:

1. *Preparation of ultra high dilutions of Bi*_*3*_*TiNbO*_*9*_* or lactose monohydrate (α-lactose) as a control.* First, trituration with lactose powder was performed. For that 1 weight part of Bi_3_TiNbO_9_ powder or lactose (DFE Pharma, Germany) and 99 weight parts of a diluent powder (lactose) were placed in a mortar and thoroughly mixed for 30 min until homogenous state. Thus, a 100-fold trituration of the original substance (Bi_3_TiNbO_9_ or lactose) was obtained. Then 1 weight part of the resulting mixed powders and 99 weight parts of lactose were thoroughly mixed in the mortar, yielding the original Bi_3_TiNbO_9_ powder or lactose powder diluted 10,000 times. Similarly, a further 100-fold dilution was prepared using lactose powder. Next, consequent dilutions in aqueous alcohol were performed. For that 1 weight part of the resulting Bi_3_TiNbO_9_-containing powder or lactose powder was diluted in 99 weight parts of 25% aqueous ethanol and shaken vigorously in a lidded vial (half filled), obtaining a further, aqueous-alcoholic dilution of Bi_3_TiNbO_9_ or lactose. Next, 1 volume part of the obtained solution and 99 volume parts of 25% aqueous ethanol were mixed by shaking vigorously in a lidded vial (half filled), obtaining the next-level centesimal aqueous-alcoholic dilution of Bi_3_TiNbO_9_ or lactose. Similarly, further dilution steps were performed using 25% aqueous ethanol solutions. 70% aqueous ethanol solution was used as diluent for two consequent dilutions preceding the final dilution that was performed in 36.7% aqueous ethanol. All subsequent dilutions comprised one part of the previous dilution and 99 parts of solvent, with intensive vibration treatment between the dilution steps. Final solution contained the mixture of 12th, 30th, and 50th centesimal dilutions. The theoretical concentration reduction level of the original Bi_3_TiNbO_9_ was 1 × 10^24^ at least. To date, physical and chemical studies have shown that highly diluted solutions are self-organizing dispersed systems in which nanoscale objects are generated^34^. Such self-organizing highly diluted substance solutions differ in properties from solvent that has undergone the same treatment. Moreover, the solution of highly diluted substances contained aggregates of initial substances with gas nanobubbles, which were preserved after repeated dilution due to the flotation effect^35^. These results are consistent with the previously described presence of the starting source materials in nanoparticulate form even at a very high dilution level^31^. All of the above means that these factors should be taken into account when calculating the concentration and discussing the physical mechanism of activity of highly diluted substances.
2. *Wetting of Bi*_*3*_*TiNbO*_*9*_* powders.* Bi_3_TiNbO_9_ powder (80 g) was wetted with 25 ml of Bi_3_TiNbO_9_ UHDs or lactose UHDs that were obtained during the previous stage or 36.7% aqueous ethanol until homogeneity was reached. Lactose UHDs or 36.7% aqueous ethanol were used as controls, since both lactose and ethanol were used at different steps of sample preparation (trituration and multiple dilutions—see stage 1). Aqueous ethanol (36.7%) was prepared from the ethanol and purified water used to prepare the aqueous-alcoholic Bi_3_TiNbO_9_ UHDs or lactose UHDs.
3. *Drying of Bi*_*3*_*TiNbO*_*9*_* powders.* The resulting wet powders were dried at + 35 °C for at least 6 h until visible evaporation of the liquid.

The following specimens were obtained using the special sample preparation process to use in the subsequent studies:

1. an intact Bi_3_TiNbO_9_ powder used as untreated control specimen;
2. Bi_3_TiNbO_9_ powder treated with 36.7% aqueous ethanol (diluent control);
3. Bi_3_TiNbO_9_ powder treated with Bi_3_TiNbO_9_ UHD (main sample);
4. Bi_3_TiNbO_9_ powder treated with lactose UHD (UHD treatment control).

Sample preparation was performed within a clean environment with all precautions taken to prevent the risk of contamination.

### Fabrication of the pre-compacted material

The obtained batches of Bi_3_TiNbO_9_ powders were calcined at 150 °C for 4 h and then passed through a 700 µm sieve. Thereafter, pre-compacted powders were prepared by the addition of 5 weight per cent of 5% aqueous polyvinyl alcohol (PVA) solution, thorough mixing and passing through a sieve.

The prepared pre-compacted powders were charged to a cylinder-shaped mold and compressed in an in-house engineered hydraulic press system under a pressure of 150 MPa to produce pre-compacted cylindrical sector pellets (50 mm in diameter and 10 mm thick).

### Specimen preparation by hot pressing

**Figure 1:**
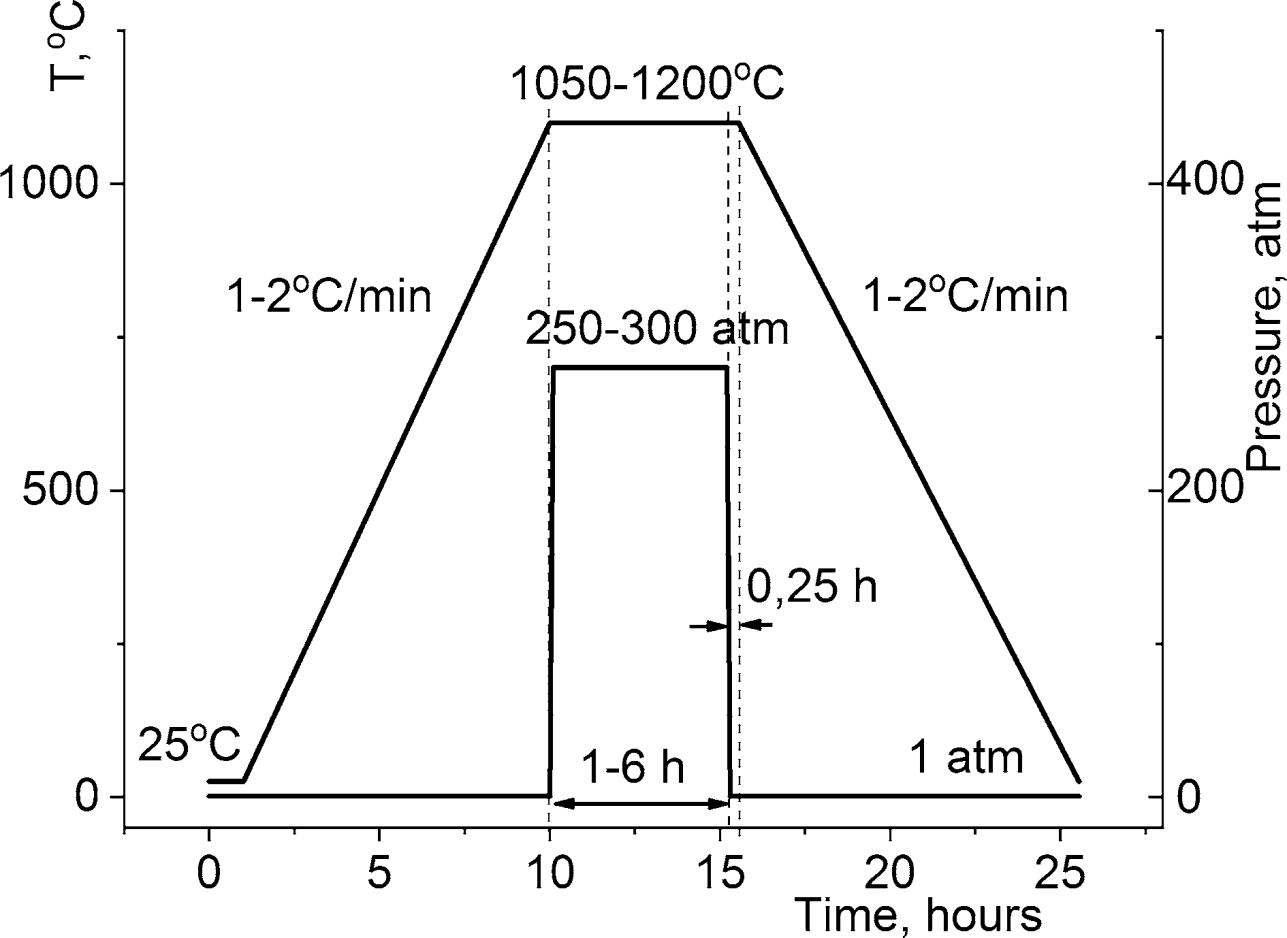
Hot pressing mode of Bi_3_TiNbO_9_ ceramics.

**Figure 2:**
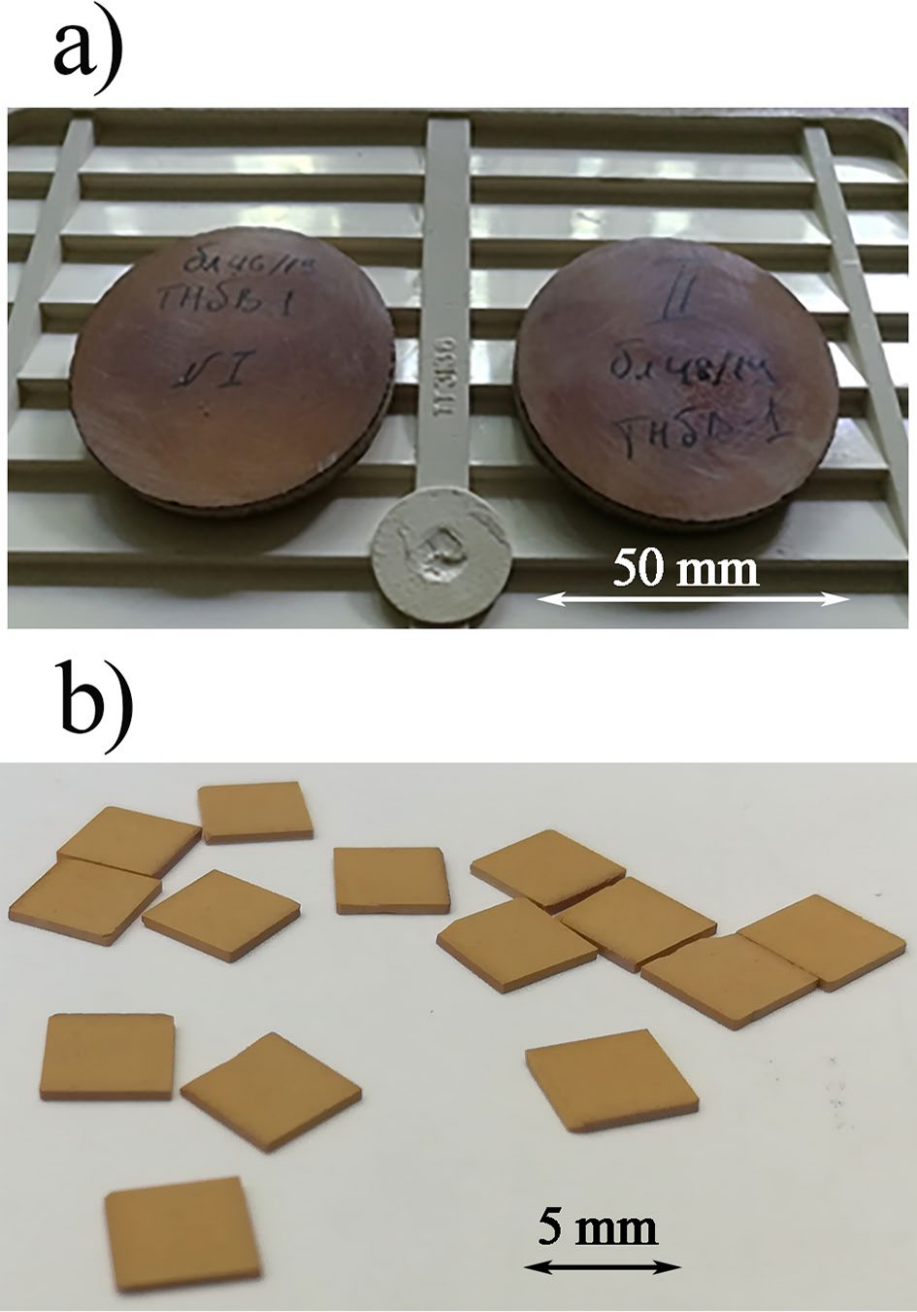
Photos of hot-pressed ceramic samples. (**a**) Appearance of Bi_3_TiNbO_9_ samples after hot pressing, (**b**) Cut plate samples for electrophysical measurements.

Each of the obtained pre-compacted specimens was individually pressure-sintered in an in-house engineered hot pressing machine using electrocorundum particles at a sintering temperature of 1423 К and pressure of 250 to 300 kgf/cm^2^ for 1 to 4 h under an air atmosphere (Fig. 1). The resulting sintered ceramic composites are shown in the photograph (Fig. 2a). The density of the ceramic samples was measured by hydrostatic weighing using a precision balance (ADAM HCB302, ADAM Equipment, United Kingdom) and constituted 97–99% of their X-ray density.

### Preparation of specimens for electrophysical studies

Hot-pressed specimens have planar textures in which the crystallographic ***c*** axis is oriented mainly along the pressing axis, i.e. perpendicularly to the basal planes of the sintered plates. Since the spontaneous polarization direction in Bi_3_TiNbO_9_ is perpendicular to the pseudo-tetragonal ***c*** axis in the crystal structure^9–11^, the strongest piezo- and pyroelectric effects should be exhibited by sections with the basal planes oriented parallel to the pressing axis as compared to sections cut along the direction parallel to the basal planes of the hot-pressed sheets.

For electrophysical measurements, the hot-pressed cylinder-shaped bulk was used to cut sections along and transverse to the direction of the basal planes (i.e. perpendicularly and parallel to the direction of the pressure applied on hot pressing). Those were cut as 0.5 × 5 × 5 mm sections (Fig. 2b), then washed with distilled water in an ultrasonic bath and dried at 673 К.

Conductive electrodes were applied on the basal planes of obtained specimen sheets by firing in Ag or Ag/Pd (70/30%) conductor pastes at 1023–1223 К for 15–30 min.

### Specimen polarization

Initially, specimens were polarized in polyethylsiloxane liquid after pre-heating at 160–180 °C and exposure to an electric field of 14 kV/mm. However, under these conditions the liquid started to boil causing the surface break-down and sample degradation. After the polarization temperature was lowered to 140–150 °C, it was still not possible to obtain stable d_33_ values. This was caused by difficulties polarizing Bi_3_TiNbO_9_ due to the high Curie temperature (T_c_ = 1180 К), and therefore a high coercive field.

In the light of these results, polarization was performed in a compressed air system (Elpa, Russia) by applying a 10 kV/mm electrical field with 15–30 min exposure at 200 °C followed by reduction of the temperature to 50–60 °C. These proved to be the optimal polarization conditions for Bi_3_TiNbO_9_ ceramics.

### X-ray diffraction studies

The phase composition of the samples was determined by X-ray diffraction with a DRON-3 automated X-ray diffractometer (AO NPP Burevestnik, Saint Petersburg, Russia) using crystalline Ge powder as an internal standard.

### Microstructure analysis

Micrographs of the surfaces of the synthesized ceramic samples sectioned with a diamond saw parallel to the direction of the applied pressure during hot pressing were obtained using a Carl Zeiss Supra 40-30-87 scanning electron microscope. Each sample was examined using two different detectors: a backscattered electron detector and a secondary electron detector.

### Piezoelectric d_33_ modulus measurements

The piezoelectric coefficient (d_33_) was measured by exposing the previously polarized ceramic specimens to varying mechanical stress using a quasi-static d_33_ meter (YE2730A, APC International Ltd., USA) at a frequency of 110 Hz at room temperature.

### Measurement of temperature- and frequency-dependent changes in dielectric permittivity (ε) and dielectric loss tangent (tan δ)

For the piezoelectric ceramic specimens, changes in dielectric permittivity (ε) with temperature or frequency as well as their dielectric loss tangent (tan δ) were measured at temperatures between 290 and 1250 К and frequency range of 25–10^6^ Hz using an E7-20 RLC meter (MNIPI, Belarus) interfaced to a computer, with a measuring voltage amplitude of 1 V. For this, a special measuring cell was used to heat and cool the specimens at 5 to 10 K/min.

### Pyroelectric coefficient measurements

Temperature-dependent changes in temperature-stimulated depolarization currents (TSDC) were measured in a short-circuit mode using a B7-30 electrometer (Amkodor-Belvar, Belarus), with the input attached to the specimen’s electrodes. The measurements were performed at a heating speed of ~ 0.1 degrees per second.

## Results

### X-ray diffraction studies

#### Phase composition of the specimens

**Figure 3:**
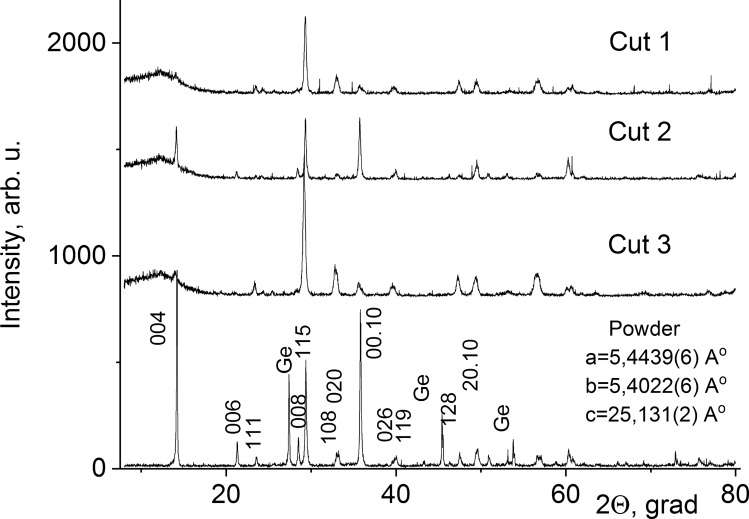
Diffraction patterns taken in Θ–2Θ geometry for ceramic powder and hot-pressed samples. Cuts 1, 3—made parallel to the pressing axis, cut 2—made perpendicular to the pressing axis. Ge crystal powder was used as an internal reference. Miller indices of the corresponding crystallographic planes are shown above reflections.

The diffraction positions and intensities of all the reflections in the X-ray diffraction patterns of the samples (Fig. 3) were consistent with the literature data on Bi_3_TiNbO_9_ [^36^, file 79–1550]. All peaks of X-Ray diffraction patterns of sintered ceramic specimens belong to the Bi_3_TiNbO_9_ phase with the structure of layered perovskite. Impurity peaks of noticeable intensity were not present. We conclude that the produced samples of all batches are essentially single-phase, they consist of a two-layer (n = 2) phase of the Aurivillius Bi_3_TiNbO_9_.

#### Bi_3_TiNbO_9_ unit cell parameters

We used high-precision measurements of Bragg angles of X-ray diffractions 2Θ (maximum measuring error: 0.02°) to get preliminary data on the symmetry and dimensions of a Bi_3_TiNbO_9_ unit cell [^36^, file 79-1550] (space group A2_1_am, *a* = 5.4398(5), *b* = 5.3941(7), *c* = 25.099(7) Å)). Here and thereafter, the number in brackets represents an approximation of the numerical value of statistical margin (± for the last significant digit). Special software (“CELREF for unit cell refinement”)^37^ made it possible to obtain diffraction patterns (see Fig. 3) which were identified within the Cmc2_1_ space group based on a rhombic unit cell with *a* = 25.18, *b* = 5.41 и *c* = 5.43 Å. This provided refined data of unit cell dimensions for Bi_3_TiNbO_9_ in the synthesized specimens. Dimensions *a*, *b* and *c* of a Bi_3_TiNbO_9_ orthorhombic unit cell in different synthesized ceramic specimens (see Table 1 and Fig. 3) were almost identical within their measuring errors, and were in agreement with the literature data on the phase [^36^, file 79-1550]. Note that the difference between our results and the literature data for the unit cell parameters of Bi_3_TiNbO_9_ clearly goes beyond the measurement errors. We believe that the difference is due to some discrepancies in the methods and conditions used to synthesize the samples.

**Table 1:** Synthesis conditions, structural and electrophysical characteristics of different series of BTN samples.

	Batch 1	Batch 2	Batch 3	Batch 4
Treatment with UHD components	An intact Bi_3_TiNbO_9_ powder	Bi_3_TiNbO_9_ powder treated with 36.7% ethyl alcohol	Bi_3_TiNbO_9_ powder treated with Bi_3_TiNbO_9_ UHDs	Bi_3_TiNbO_9_ powder treated with lactose UHDs
Rhombic unit cell parameters	*a* = 25.184(8)Å *b* = 5.412(1) Å *c* = 5.436(1) Å	*a* = 25.171(8)Å *b* = 5.411(1) Å *c* = 5.432(1) Å	*a* = 25.174(8)Å *b* = 5.409(1) Å *c* = 5.436(1) Å	*a* = 25.163(8)Å *b* = 5.406(1) Å *c* = 5.433(1) Å
Dielectric permittivity ε	102.6(3.4)	99.0(3.7)	98.2(4.6)	98.6(4.9)
Piezoelectric coefficient d_33_, pC/N	10.1(0.2)	10.6(0.4)	12.7(1.1)	10.7(0.2)
Pyroelectric coefficient p^σ^, nC/(cm^2 ^K)	0.75(3)	0.80(2)	0.96(6)	1.01(3)

Data are presented as average, n = 10–18 repeated measurements. The numbers in brackets represent an approximation of the numerical value of statistical margin (± for the last significant digit).

#### Texturing of the hot-pressed ceramics

Figure 3 shows the diffraction patterns recorded in Θ–2Θ geometry from the surfaces of plates oriented differently to the basal plane of the hot-pressed bulk. In Sect. 2, parallel to the basal plane, intense (001) reflections are observed, which are considerably attenuated or even extinct in perpendicular cuts (cuts 1 and 3). This indicates that with hot pressing, the (001) planes of BTN crystallites tend to align themselves perpendicular to the pressing direction, contributing to the formation of an ordered microstructure in ceramics. Thus, the obtained specimens have planar textures. The texturing plane is defined by the direction of the force imposed on the plane of a hot-pressed bulk specimen.

A quantitative estimation of the texture degree of the ceramics was performed using Lotgering’s formula f = (P − P_o_)/(1 − P_o_), where P = ΣI(00l)/ΣI(hkl) for textured samples and P_o_ is P for a randomly oriented sample^38^. It was found that the value of the degree of texturing f equaled approximately 0.70, which indicates a very high degree of grain orientation. Variations in the value of f between samples of different series did not exceed the measurement error (5%). From these data it follows that the differences in the degree of texturing of samples from different batches are insignificant.

#### Microstructure of ceramics

**Figure 4:**
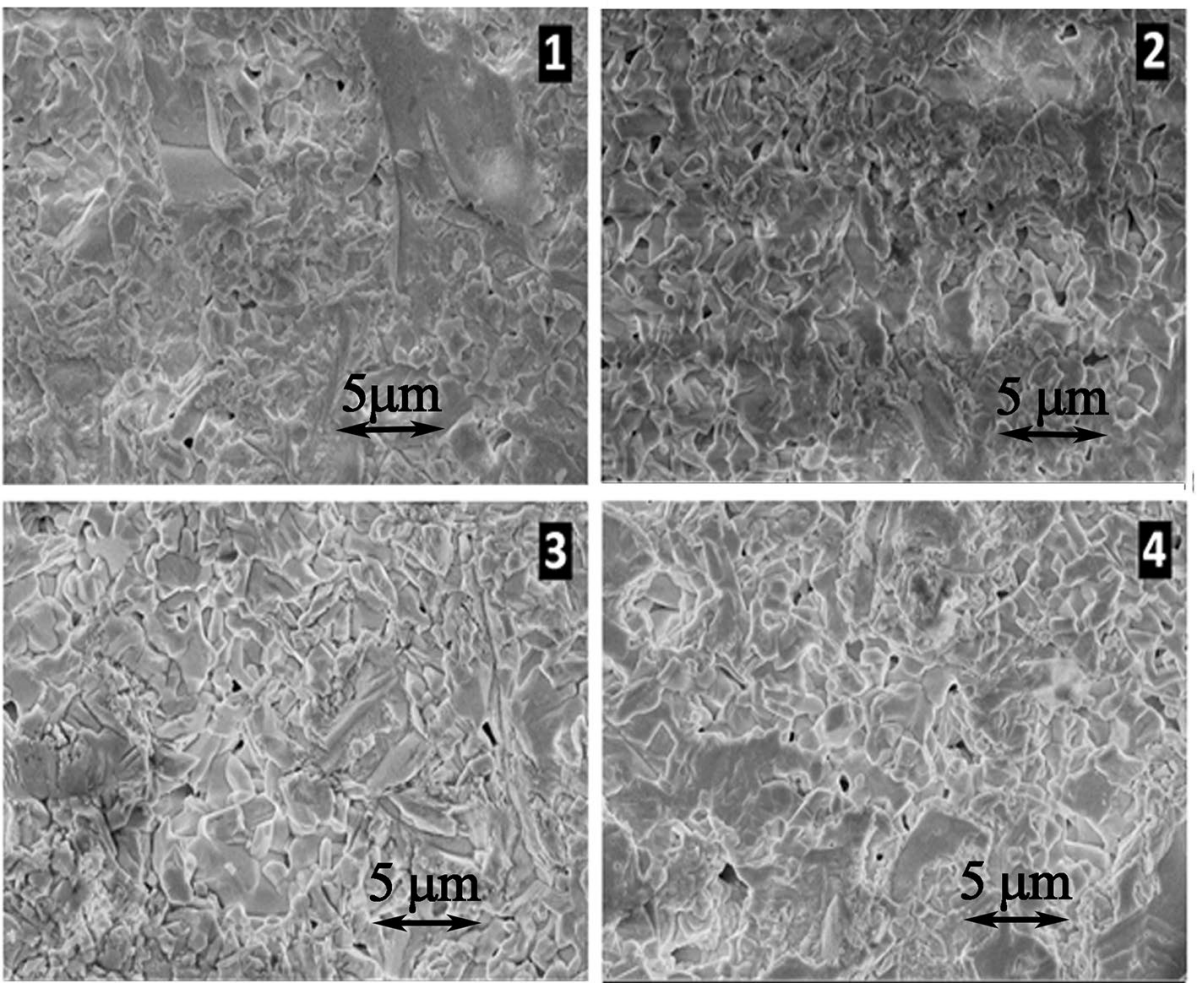
SEM images of the surfaces of ceramic samples (backscattered electron detector imaging) synthesized from Bi_3_TiNbO_9_ powders:** 1**—intact;** 2**—treated with 36.7% aqueous ethanol;** 3**—treated with Bi_3_TiNbO_9_ UHD;** 4**—treated with lactose UHD.

Photos of all types of specimens were obtained using scanning electron microscopy (see Fig. 4). It can be seen that the grain size of the ceramics is ~ 1 μm. No qualitative differences in the microstructure of samples of different batches were observed. Therefore, the differences in the properties of ceramics of different series are probably due to some modifications of the atomic-nanocrystalline structure that occur during the UHD processing.

### Piezoelectric properties

Polarized specimens have been shown to exhibit a pronounced piezoelectric effect. In the course of this study, the piezoelectric properties were found to significantly deteriorate with silver electrodes applied. This is associated with uncontrolled silver diffusion into the ceramic structure and an increase in the reach-through conductivity. Therefore, the key measurements were performed using Ag/Pd electrodes. For better validity of the method, the specimen sheets were positioned inside the measuring system (YE2730A d_33_ meter from APC International, Ltd., USA), with their sides being flipped in between measurements (i.e. manually shifting the relative direction of polarization of the piezoceramics sheet). The data are presented below as absolute values.

**Figure 5:**
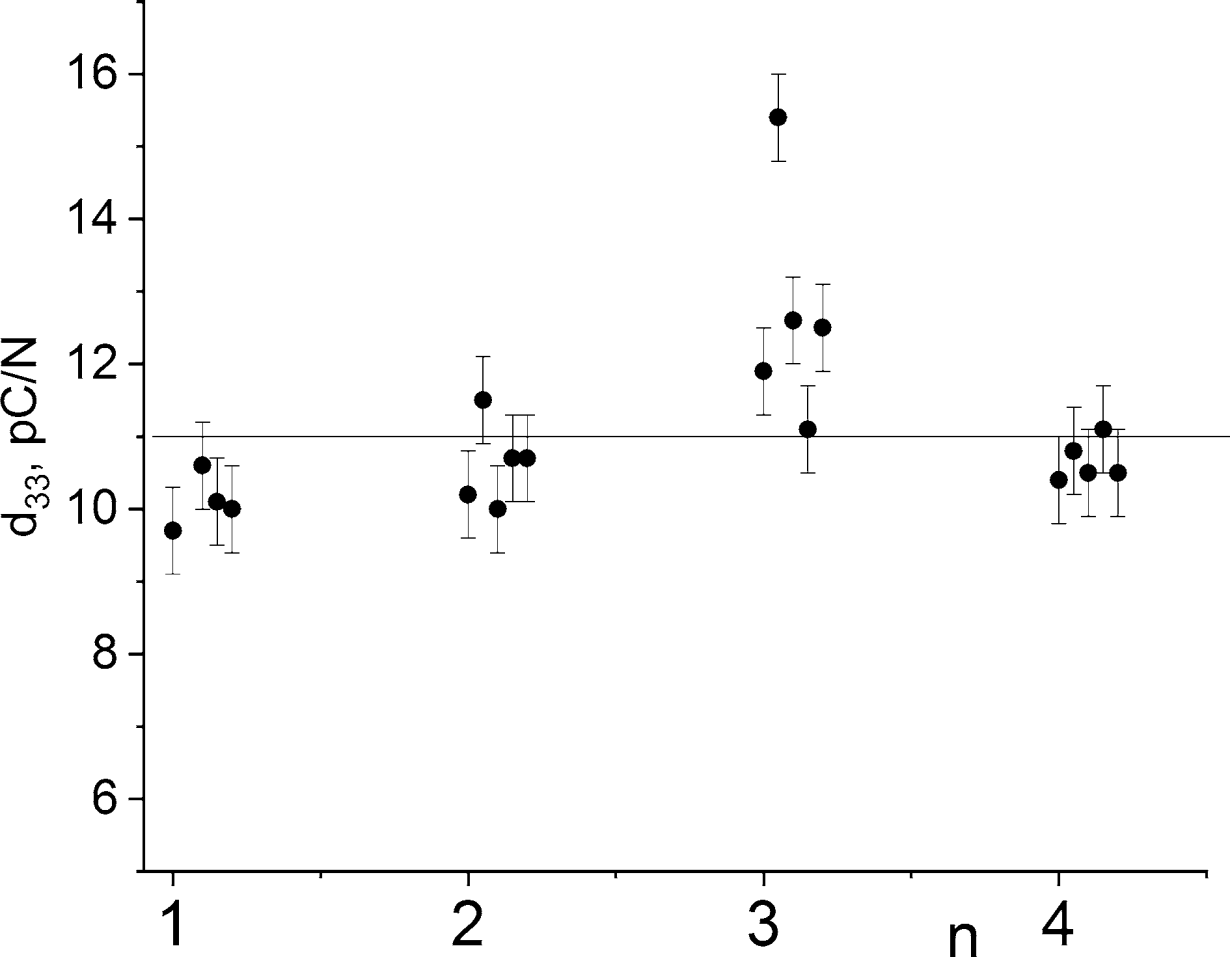
Piezoelectric coefficient d_33_ values for Sect. 1 of hot-pressed Bi_3_TiNbO_9_ samples of different series. A horizontal line corresponds to the d_33_ value averaged over all measurements, equal to 11.1 pC/N. Sample modifications: (1) an intact Bi_3_TiNbO_9_ powder (2) Bi_3_TiNbO_9_ powder treated with 36.7% ethyl alcohol; (3) Bi_3_TiNbO_9_ powder treated with Bi_3_TiNbO_9_ UHDs; (4) Bi_3_TiNbO_9_ powder treated with lactose UHDs.

In Table 1 and Fig. 5 are presented the results of d_33_ measurements performed for polarized specimens across the batches, where each value provided is an averaged result over 10 to 18 measurements for the same specimen.

Plate-like specimens with the basal planes oriented along the hot pressing direction have higher piezoelectric coefficient values of ~ 10 to 15 pC/N (with the average value for all specimens of 11.1(5) pC/N), as compared to cuts of other orientations. This increase in the d_33_ value is obviously due to the obtained ceramics being textured and favorable alignment of the crystallites with respect to the direction of spontaneous polarization. Noteworthy, the average d_33_ value obtained for specimens from batch 3 (12.7 pC/N) is significantly higher than the average (11.1 pC/N) d_33_ measured for all batches (Fig. 5).

As described in the Materials and methods section, samples were polarized 200 °C. To determine depolarization temperature ceramic specimens were heated above 200 °C at 50 °C increments. After each step specimens were cooled to room temperature and piezoelectric coefficient (d_33_) was measured. It was found that the d_33_ values of the specimens were unchanged upon heating at temperatures up to 700 °C (973 К). Thus, depolarization of the ceramics did not occur until this temperature*.*

### Measurements of dielectric permittivity and dielectric loss tangent

**Figure 6:**
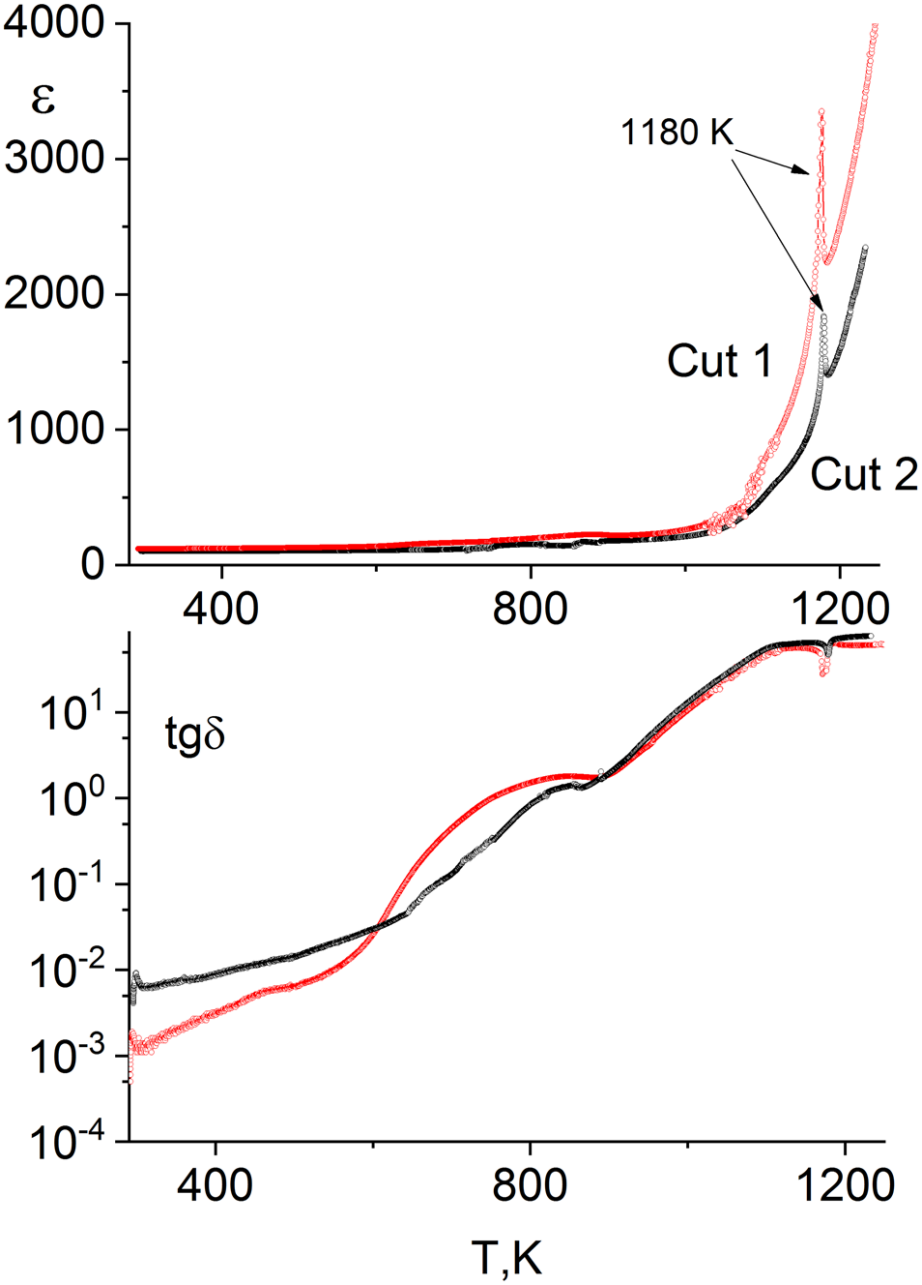
Temperature dependences of the permittivity (ε) and the dielectric loss tangent (tan δ) of hot-pressed sections of BTN plates at the frequency of the measuring field of 1 MHz: the upper section is the permittivity (ε), the lower section is the dielectric loss tangent (tan δ).

**Figure 7:**
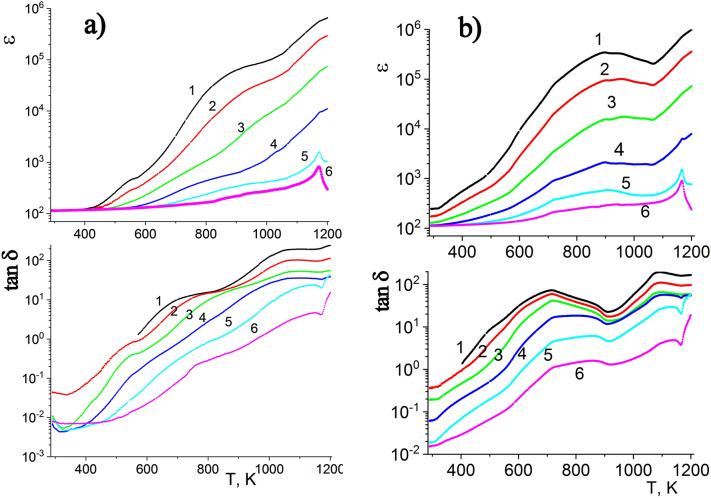
Temperature-frequency dependences of the dielectric permittivity (ε) and dielectric losses (tan δ) of samples of series 1(**a**) and 3(**b**), measured at the temperature range 290–1220 K and at different frequencies: 1—0.025; 2—0.120; 3—1, 4—10, 5—100 and 6—1000 kHz.

**Figure 8:**
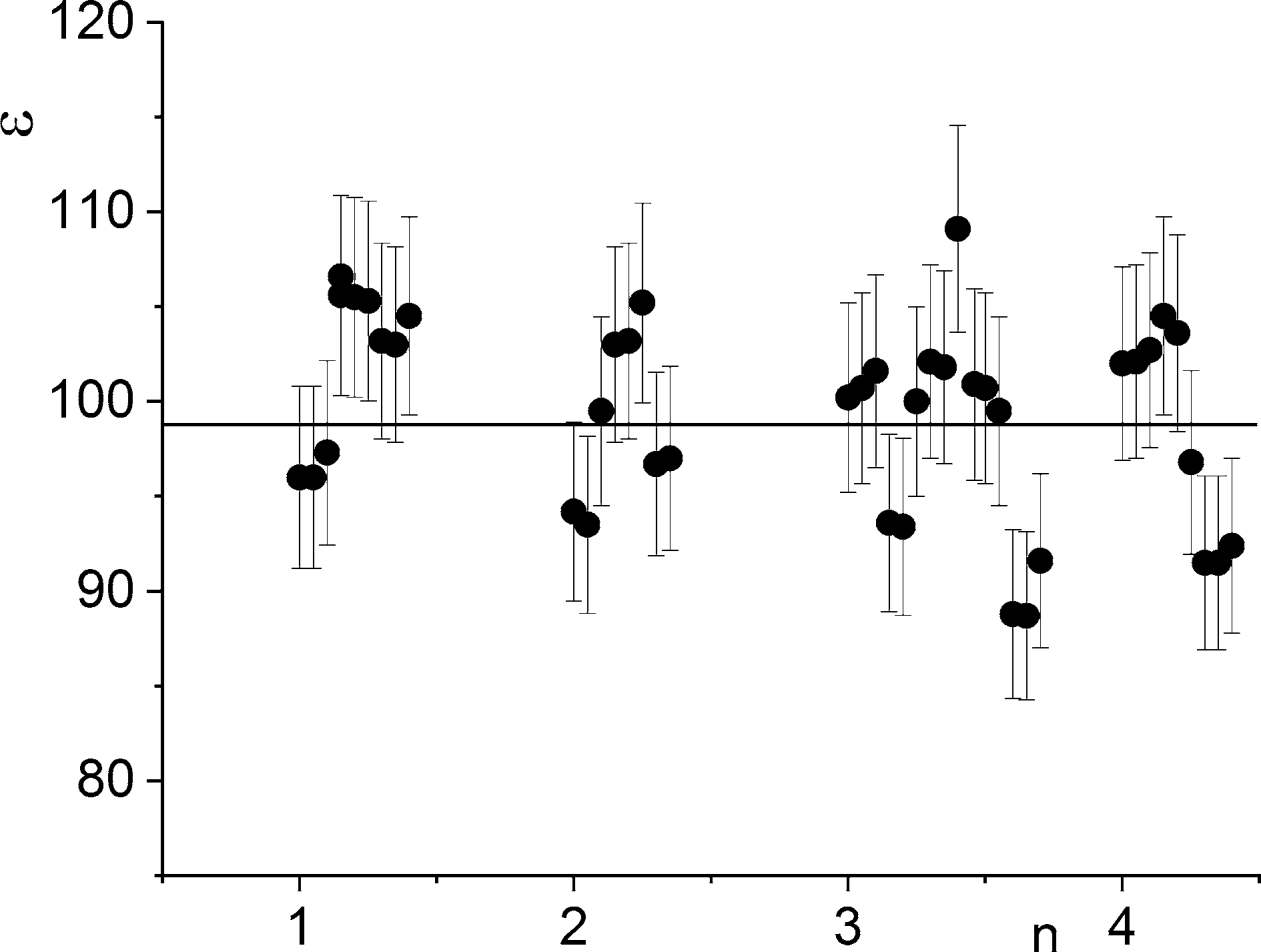
The dielectric constant (ε) of samples of different series of hot-pressed ceramics Bi_3_TiNbO_9_ at room temperature, measured at a frequency of 1 kHz (the horizontal line indicates the average value ε = 98.8 for all measurements). Sample modifications: (1) an intact Bi_3_TiNbO_9_ powder (2) Bi_3_TiNbO_9_ powder treated with 36.7% ethyl alcohol; (3) Bi_3_TiNbO_9_ powder treated with Bi_3_TiNbO_9_ UHDs; (4) Bi_3_TiNbO_9_ powder treated with lactose UHDs.

The obtained data are shown in Figs. 6, 7 and 8. Clear maxima are observed for dependencies ε(T) at T_c_ = 1180 К (Figs. 6, 7), apparently caused by the ferroelectric phase transition occurring in the BLSF phase contained by the specimens, with the temperature at the maximum corresponding to the Curie temperature (T_c_) of the phase.

No shifts are observed in the position of the ε maximum at T_c_ with changes in the frequency of the measuring field. This maximum degenerates into a point of inflection under the effect of conductivity at lower frequencies. A minimum is identified for dependencies tan δ(T) at T_c_, while at temperatures ~ 50 К lower than T_c_ there is a maximum apparently caused by domain movement.

Consistent with texturedness of the fabricated ceramics, the dielectric permittivity value depends on the orientation of the ceramic section relative to the pressing direction. Dielectric permittivity maxima are most pronounced in cut 1 of plate sample, which is in line with the direction of spontaneous polarization P_s_. The following dielectric permittivity value is obtained at around room temperature: ε = 100, and it increases to ε = 3350 at T_c_. The spontaneous polarization vector lies in the plane of cut 2 of plate sample. At the Curie temperature, the maxima of ε(T) dependencies are two times lower than the ε of cut 1 of plate sample (Fig. 6).

Figure 7 shows the temperature-frequency dependences of the permittivity ε and dielectric losses tan δ of samples of series 1 and 3, measured in the temperature range of 290–1220 K and the frequency range of 25–10^6^ Hz. The dependencies ε(T) and tan δ(T) of samples of different series are qualitatively and quantitatively similar to each other. In addition, the ε(T) and tan δ(T) curves have maxima in the ranges of 890–920 and 715–860 K, respectively. In previous reports^18,19,23,26^, such maxima were tentatively attributed to relaxation processes related to the presence of mobile oxygen vacancies in the structure of the phase.

#### Dielectric permittivity (ε) values

The dielectric permittivity values obtained at room temperature and at kHz frequency range are provided in Table 1 and Fig. 8, with each value being the result of averaging over 10 successive measurements. The dielectric permittivity (ε) values are nearly identical among the different batches of specimens. The variance of the measured value at 1 kHz at room temperature (Fig. 8) is almost within the ε measuring error. The variability observed in the obtained values is due to differences in ε measured for different specimens as well as to random ε measuring errors, which are mainly accounted for by the measuring error in the values of electrode areas of the specimens (~ 5%).

### Pyroelectric coefficient measurements

**Figure 9:**
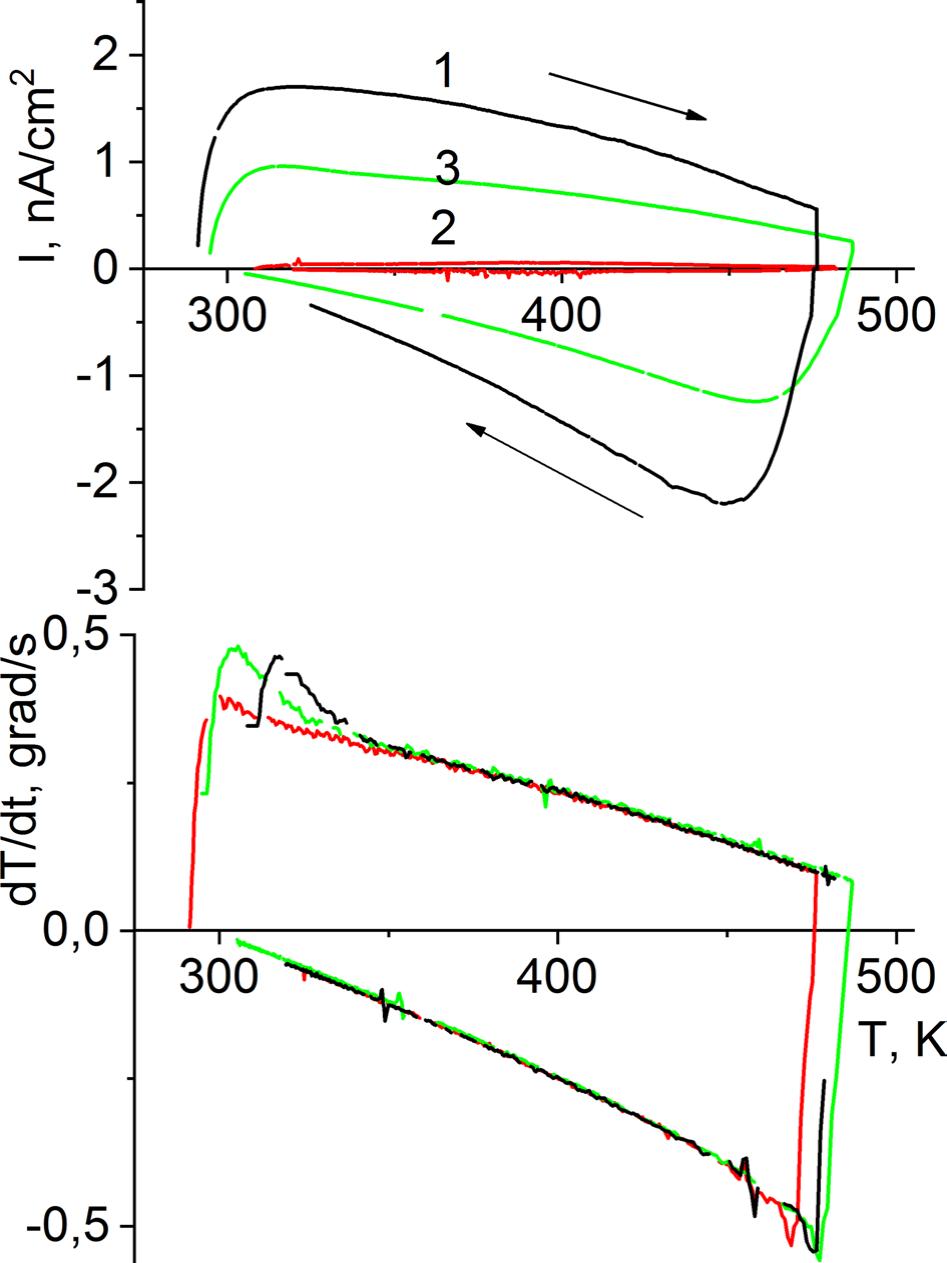
Temperature dependences of currents of thermally stimulated depolarization (TSDC) and heating rate of hot-pressed BTN cuts 1, 2, and 3.

The results of the measurements performed for hot-pressed Bi_3_TiNbO_9_ ceramic specimens are shown in Fig. 9 as plots of TSDC changes with temperature normalized for electrode areas (S) and rate of temperature variation (dT/dt) observed on heating and cooling the specimen. As shown in the figure, the changeover from heating to cooling reverses the sign of the measured current, which according to equation I_p_ = p^σ^S(dT/dt) points to pyroelectric nature of the current. Therefore, as described by the famous formula—I_p_ = p^σ^S(dT/dt)^39^, normalized TSDC values are the p^σ^ pyroelectric coefficient at constant mechanical stress σ.

**Figure 10:**
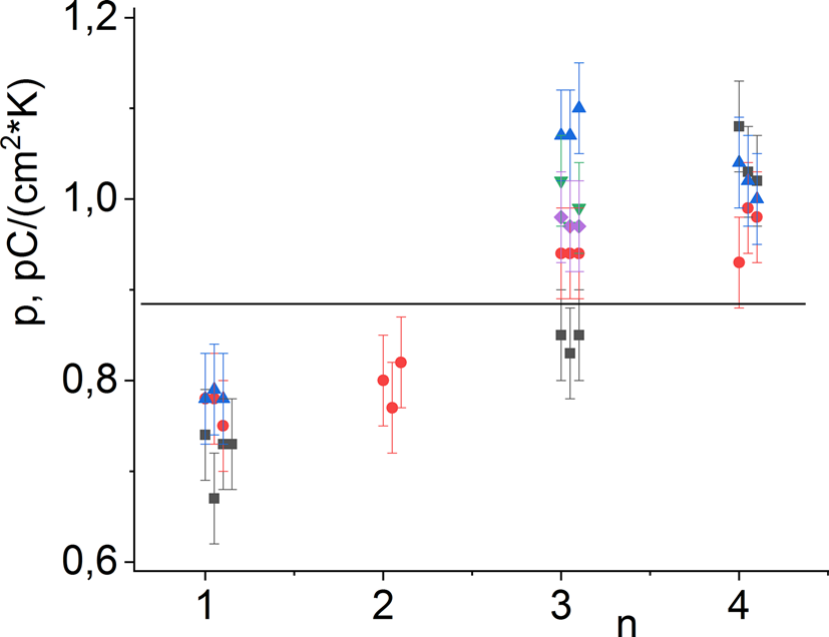
The values of the pyroelectric coefficient measured for samples of different series of the hot-pressed Bi_3_TiNbO_9_ ceramics (the horizontal line indicates the average pyroelectric coefficient p^σ^ = 0.88 nC/(cm^2^·K) for all measurements). Sample modifications: (1) an intact Bi_3_TiNbO_9_ powder (2) Bi_3_TiNbO_9_ powder treated with 36.7% ethyl alcohol; (3) Bi_3_TiNbO_9_ powder treated with Bi_3_TiNbO_9_ UHDs; (4) Bi_3_TiNbO_9_ powder treated with lactose UHDs.

Figure 10 graphically illustrates the pyroelectric coefficient values obtained at room temperature, demonstrating variability of measured p^σ^ values from specimen to specimen, between different batches and within each batch. Notably, the average p^σ^ values obtained for specimens 3 and 4 (0.96 and 1.01 nC cm^−2^ degree) are 9–15% higher than the average p^σ^ (0.88 nC·cm^−2 ^K) for all the measurements.

## Discussion

Four batches of Bi_3_TiNbO_9_ perovskite layer structured ceramic specimens, with measured densities corresponding to 97–99% of their X-ray density, were prepared by hot pressing from Bi_3_TiNbO_9_ powders previously exposed to different treatments obtained using the UHD process. X-ray diffraction studies indicated that the studied piezoceramic specimens consisted of the Bi_3_TiNbO_9_ phase; the specific parameters of a BTN orthorhombic unit cell determined for specimens from different batches were nearly identical. The specimens had an ordered microstructure and were of planar texture. The physicochemical properties demonstrated anisotropy indicative of definite texturedness of the ceramics on X-ray diffraction patterns.

The Bi_3_TiNbO_9_ phase was selected for research due to considerable practical interest of this phase for creating high-temperature piezoelectric materials (because of its very high Curie point T_c_ = 950 °C). An improvement in the piezoelectric properties of Bi_3_TiNbO_9_ ceramics to d_33_ = 17 pC/N was achieved by adding Ce atoms to this phase^23^. However, d_33_ value for pure ceramics was significantly lower (2.3 pC/N) than in our work. Similar findings were reported by Peng et al.^21^. In this study, an increase in d_33_ value was achieved by adding Ta/W. A significantly lower value (2.5 pC/N) was obtained for the pure phase of Bi_3_TiNbO_9_ as well.

It is known that for unmodified BTN ceramics made without hot pressing, the value of the piezoelectric coefficient d_33_ is 6 pC/N^25^. After hot pressing the d_33_ values for Bi_3_TiNbO_9_ powder modified with UHD of Bi_3_TiNbO_9_ turned out to be two times higher than for unmodified BTN ceramics made without hot pressing and coincides with the value of the piezoelectric coefficient for this compound with a small addition (doping) of tungsten^1^. Taking into account the extent of the improvement, this technology could be even more promising for the materials with initially high piezoelectric coefficient. The extension of such studies is obviously necessary.

Together with the main sample (Bi_3_TiNbO_9_ powder treated with Bi_3_TiNbO_9_ UHDs), hot pressing was applied to a control (intact Bi_3_TiNbO_9_) which was not saturated with UHDs and was manufactured according to the standard hot pressing method. All of that was done in order to understand what exactly was the contribution of the serial dilution method. For the intact Bi_3_TiNbO_9_, the average value of the piezoelectric coefficient d_33_ was about 10 pC/N; for technological controls—Bi_3_TiNbO_9_ modified with UHDs of lactose or 36.7% aqueous ethanol—approximately 10.5 pC/N and for sample modified with Bi_3_TiNbO_9_ UHDs—about 12 pC/N. It can be noted that the controls and main sample differ in average piezoelectric coefficient by more than 20%, similar differences were also observed for the values of the pyroelectric coefficient. The influence on this parameter in one of the technological controls (Bi_3_TiNbO_9_ modified with UHDs of lactose) is also noticeable (Fig. 10). We can assume that this might reflect the non-specific effect of the solvent subjected to sequential dilutions on the properties of the initial piezoelectric material powder. Thus, judging by the absence of a visible difference in the microgranular structure of the obtained ceramics and by the implementation of three types of controls, we can assume that the observed change in properties is associated with the use of the UHD technology, and not with the use of the hot pressing method. Measurements of characteristics of different ceramic samples of the same batch provided results with reproducibility within 3% (Figs. 5, 8). Each value given in the Table 1 is an average of 10 measurements of the same sample. Thus, close values of the measurement results for the control samples indicate the reproducibility of the results and the method.

The main interest in the application of sequential dilution technology is that there are unexpected effects on the material properties. Studying the applications of UHD technology in biology, it was found that the addition of UHD of antibodies could cause the change in conformation of the target protein^40^. Also, some authors report that a substance may completely change its properties when tightly surrounded by certain structures^41,42^. As mentioned earlier, UHD process leads to formation of nanostructures and nanobubbles^31–33^. Probably, the presence of such bubbles surrounding the initial BTN powder and the change in the parameters of the solvent caused by them can affect its properties. We suggest that such an effect may be associated with a change in the order of formation of the microcrystalline structure due to point defects (similar to doping in ionic crystals) arising during a complex of mechanical interactions, as well as changes in conditions and rearrangement of the energy balance during the formation of the crystal lattice.

## Conclusions

A novel method is proposed for processing oxide powders with the structure of Aurivillius phases of Bi_3_TiNbO_9_ which results in an increase in the piezoelectric modulus of high-temperature ceramics produced from them. This method is based on the ultra high dilution of the original powders. It is easy to use, does not require expensive doping with high-purity materials, and does not add significant costs.

Ceramics made from powders pretreated with UHD have higher values of the pyroelectric coefficient p^σ^ (0.96–1.01 nC/(cm^2 ^K)) compared to control samples that were also subjected to hot pressing (0.75–0.8 nC/(cm^2 ^K)). The piezoelectric modulus d_33_ of the sample pretreated with UHD has a value of 12.7 pC/N, for the rest of the various control samples it lies within 10.1–10.7 pC/N. It is important to note that these values remained stable up to a depolarization temperature of 973 K.

The obtained characteristics of the studied ceramics make it potentially suitable for high-temperature applications, in particular, as a material for electro-acoustic transducers and/or sensors operating at high temperatures. Thus, the application of the serial dilution method makes it possible to expand the scope of applicability of piezoelectric materials.
